# *Lactobacillus plantarum* improves LPS-induced Caco2 cell line intestinal barrier damage via cyclic AMP-PKA signaling

**DOI:** 10.1371/journal.pone.0267831

**Published:** 2022-05-31

**Authors:** Chen-Xiang Wei, Ju-Hua Wu, Yue-Hong Huang, Xiao-Zhong Wang, Jian-Ying Li

**Affiliations:** 1 Department of Gastroenterology and Fujian Institute of Digestive Disease, Fujian Medical University Union Hospital, Fuzhou, Fujian Province, P.R. China; 2 Digestive Endoscopy Center, Longyan First Affiliated Hospital of Fujian Medical University, Longyan, Fujian Province, P.R. China; University of Illinois at Chicago, UNITED STATES

## Abstract

*Lactobacillus plantarum* (LP) has been shown to exhibit protective effects on intestinal barrier function in septic rats, although the regulatory mechanism has not been established. We determined whether LP imparts such protective effects in a lipopolysaccharide (LPS)-induced Caco2 cell monolayer model and whether cAMP-PKA signaling is the underlying mechanism of action. The cyclic adenosine monophosphate (cAMP) agonist, forskolin (FSK), and the protein kinase A (PKA) inhibitor, HT89, were used to study the protective effect of LP on the destruction of the tight junction (TJ) structure of cells treated with LPS and the corresponding changes in cAMP-PKA signaling. Our experimental results demonstrated that LP promoted the expression of TJ proteins between Caco2 cells after LPS treatment, and increased the electrical barrier detection (TEER) between Caco2 cells. Moreover, transmission electron microscopy (TEM) revealed that the TJ structural integrity of cells treated with LPS + LP was improved compared to cells treated with LPS alone. In addition, our findings were consistent between the FSK and LP intervention group, while HT89 inhibited LP influence. Taken together, our results indicate that LP has an improved protective effect on LPS-induced damage to the monolayer membrane barrier function of Caco2 cells and is regulated by the cAMP-PKA pathway.

## Introduction

Lipopolysaccharide (LPS), a major component of the outer membrane of Gram-negative bacteria, activates cells, such as macrophages, endothelial cells, and epithelial cells, causing host cells to produce cytokines and inflammatory mediators [1, 2]. The inflammatory response is a defense mechanism against infection. Indeed, a systemic inflammatory response, such as occurs with sepsis, leads to multiple organ failure or death [3]. Sepsis is a clinical syndrome characterized by infection-induced systemic inflammation and circulatory impairment [4, 5].

Several studies have shown that 1–50 μg/ml of LPS triggers rapid death and apoptosis of different cell types; however, these studies have not accurately defined a physiologic concentration of LPS that ensures biologic activity [6, 7]. Panaro et al. (2016) demonstrated that 1 μg/ml of LPS instigates an inflammatory response in Caco2 cells without causing cell death. Caco2 cells are derived from human colon adenocarcinoma cells and have many similarities with small intestine absorptive cells. Caco2 cells are often used in *in vitro* experiments to study intestinal barrier function [8].

With recent changes to the theory of microbial symbiosis, many scientists have turned their attention toward the impact of the intestinal flora on critical diseases [9, 10]. Multiple human and animal studies have confirmed that impaired intestinal mucosal barrier function initially causes bacterial translocation, followed by the development of sepsis [11]. Systematic research of intestinal flora is often based on the screening of clinical metabolomics and bioassay analyses [12]. *Lactobacillus plantarum* (LP) has a decisive role in promoting the stability of the entire intestinal microecology [13]. Martin-Venegas et al. (2017) co-cultured LP and Caco2, and demonstrated that LP with a multiplicity of infection (MOI) of 10 interferes with Caco2 cells. The origin of the concept of MOI is based on the ratio of the number of phages to bacteria at the time of infection. In our experiments, MOI was the ratio of LP to Caco2 cells [14].

The physiologic barrier of intestinal epithelial cells involves tight junctions (TJs) between cells and is integral in the mechanical intestinal mucosal barrier [15]. Tightly connected molecules form adjacent cell junction complexes that are mainly composed of multiple transmembrane proteins, including occludin, claudins, junctional adhesion molecules (JAMs), closed loop proteins, zonula occludins (ZOs 1, 2, and 3), and a protein scaffold [16]. The redistribution of TJ proteins can cause damage to TJ barriers in the microdomains of TJ membranes in sepsis [17]. LP may improve epithelial barrier function by inhibiting the decrease in TJ proteins [18].

The phosphorylation of claudin-3 by protein kinase A (PKA) is thought to be a result of oxidative stress damage to gastric epithelium permeability, leading to a decrease in TJ integrity [19]. Therefore, cyclic adenine monophosphate (cAMP)-PKA signaling may affect TJs by regulating proteins that comprise the TJs. Forskolin (FSK) and HT89 are activators of cAMP and inhibitors of PKA, respectively, and are widely used in cAMP-PKA pathway experiments [20, 21].

Our study confirmed the protective role of LP on the intestinal mucosal barrier during LPS treatment and further determined whether LP is related to cAMP-PKA pathway regulation.

## Materials and methods

### Cell culture

The Caco2 cell line was purchased from Shanghai Kelei Biotechnology Co., Ltd. (Shanghai, China). Caco2 cells were cultured in Dulbecco’s modified Eagle medium (DMEM; Hyclone, Logan, UT, USA) with 10% fetal bovine serum (PAN, Aidenbach, Bavaria, Germany) in a 5% CO_2_ humidified incubator at 37°C. Culture medium was changed every 2 days.

### LP culture and colony count

LPCGMCC 1258 dry powder (Shanghai Nuode Company, Shanghai, China) was added to MRS broth (Qingdao Haibo Biological Company, Qingdao, China) and incubated at 37°C on a constant temperature shaker at 250 rpm for 24 h. After an overnight incubation, the bacteria were centrifuged at 5000 × *g* for 10 min at 4°C, washed with cold phosphate-buffered saline (PBS), and resuspended in cold PBS to obtain a final bacterial concentration of 1 × 10^8^ CFU/ml.

### Caco2 cell and bacteria co-culture

After 2 days of Caco2 cells in culture, LP was added to the culture medium at a concentration based on the calculated number of Caco2 cells, diluted to a cell-to-bacterial colony ratio of 2:1 (MOI = 0.5). LP (soluble in DMEM) can be added directly to the culture medium, but must be shaken well. MOI = 0.5 should be assured and the number of cells cultured for 2 days should be the same.

### Reverse transcription-quantitative polymerase chain reaction (RT-qPCR)

Total RNA was isolated from mouse tissues using the Trizol reagent (Invitrogen, Carlsbad, CA, USA), cDNA synthesis was conducted with a cDNA synthesis kit (Thermo, Waltham, MA, USA). SYBR Green (Roche, Basel, Switzerland) was used in qRT-PCR by a 7,500 Real-Time PCR instrument (Applied Biosystems). Primer sequences of target genes were obtained by database primer design as follows: β-actin sequence, (F, CACAGACCTCGCCTTGCC;R, TGACCCATGCCCACCATCAC); TNF-α sequence, (F, ACTGAAAGCATGATCCGGGAC; R, GGGGCCGATCACTCCAAAG); and IL-6 sequence, (F, GTGAAAGCAGCAAAGAGGCAC; R, GGCTTGTTCCTCACTACTCTCAA). The amplification program was set to denaturation at 95°C for 15 sec, annealing at 55°C for 30 sec, and extension at 72°C for 30 sec, with a total of 40 cycles.

### Enzyme-linked immunosorbent assay (ELISA)

Cell-secreted TNF-α and IL-6 were measured with the corresponding ELISA kits (R&D Systems, Minneapolis, MN, USA) according to the manufacturer’s instructions. After intervention, cell supernatant was absorbed and centrifuged at 3000 RPM at 4°C for 10 min to remove particles and polymers. Remove the lath from the aluminum foil bag after equilibration at room temperature for 20 min. Fifty microliters of standard at different concentrations were added to each standard well. Ten microliters of sample was added to each sample well, then the sample was diluted to 40 μL; sample was not added to the blank wells. Horseradish peroxidase (HRP)-labeled antibody (100 μL) was added to each well except the blank well. The reaction holes were sealed with sealing plate film and incubated in a 37°C water bath or thermostat for 60 min. The liquid was discarded, dried with washing solution, which was then added to each well, rested for 1 min, swing the washing solution, dried with washing solution, and the plate was washed 5 times. Substrate A and B (50 μL) was added to each well and incubated at 37°C for 15 min in the dark. Stop solution (50 μL) was added to each well and 15 min later the OD value of each well was measured at 450 nm. A standard curve was drawn using the standard concentration as the abscissa and the corresponding OD value as the ordinate on an Excel worksheet to draw a linear regression curve of the standard and the concentration value of each sample was calculated according to the curve equation.

### Western blot

We extracted cellular proteins and utilized a bicinchonic acid (BCA) working solution (Beyotime, Shanghai, China) to determine the protein concentration. Equal protein concentrations per sample were loaded onto a sodium dodecyl sulfate-polyacrylamide electrophoresis gel (SDS-PAGE) and loaded proteins were separated via electrophoresis for 90 min (70 V for 30 min and 110 V for 60 min). Protein was transferred to a nitrocellulose membrane, blocked for non-specific antibody labeling, and incubated with antibodies against the following cell junction markers overnight at 4°C: anti-claudin-1 (71–7800; Thermo, Waltham, MA, USA); anti-occludin (71–1500; Thermo, Waltham, MA, USA); anti-JAM-a (ab180821; Abcam, Waltham, MA, USA); and anti-ZO-1 (QG215365; Thermo, Waltham, MA, USA). To visualize protein bands, the membranes were then washed and exposed to enhanced chemiluminescence (ECL) reagent (Beyotime, Shanghai, China) for 1 min, placed on a BIO-RAD imager (Bio-Rad, Hercules, CA, United States), and automatically exposed for imaging.

### Transepithelial/endothelial resistance (TEER)

To measure TEER, 1.8 × 10^4^ ml of Caco2 cells were seeded per well in the upper chamber of Transwell 24-well suspension culture plates (Corning, Corning, NY, USA) with 0.4-mm pores. Then, 200 μl of culture medium was added to the upper chamber, and 1 ml of medium was added to the lower chamber. After 2 days, resistance was measured with an EVOM cell potentiometer (Millipore, Billerica, MA, USA). The resistance values in each of the three directions were measured once, and the average value (Ω) was recorded. The product of the resistance and the effective area is the TEER value.

### Immunofluorescence

To fix the cells for immunofluorescence, 4% paraformaldehyde in PBS was applied to the cells at room temperature, followed by incubation with ZO-1 primary antibody (Thermo, Waltham, MA, USA) at 4°C overnight. The next day, the cells were incubated with a green fluorescent secondary antibody (Abcam, Waltham, MA, USA) at room temperature for 1 h (protected from light). Following incubation, the cells were washed 4 times in PBS, and rotated on a shaker at 70 rpm for 5 min per wash (protected from light). Next, a small drop of glycerol with an anti-quenching agent was applied to a slide glass, the glass slide with cells was cover-slipped, and the slide was placed face down and observed under a fluorescence microscope.

### Transmission electron microscopy (TEM)

Cells were fixed on a slide *in situ* using a glutaraldehyde-arsenic acid double-fixation method with a pure epoxy 618 embedding agent applied and allowed to dry at varying temperatures as follows in preparation for TEM: 35°C for 24 h; 45°C for 12 h; and 60°C for 2–3 days). The specimen was then processed in semi-thin sections and positioned for additional processing. The tissue was then cut into ultra-thin sections and stained with uranium acetate for 20 min. The tissue was washed with distilled water, lead citrate for 5–10 min, and again with distilled water, and observed and imaged under a Tecnai transmission electron microscope (FEI; Hillsboro, OR, USA).

### Statistical analysis

All data were statistically analyzed using SPSS 22.0 and GraphPad Prism 7.0 software (GraphPad Software, SD, USA). The comparison of means between groups was performed by one-way analysis of variance (ANOVA) or a t-test. P < 0.05 indicated statistical significance.

### Ethical approval

This article does not contain any studies with human participants or animals performed by any of the authors.

## Results

### The appropriate ratio of LP-to-Caco2 cells is 1:2 (MOI = 0.5) because LP with a MOI = 0.5 promotes the expression of TJ proteins (claudin-1 and occludin) in Caco2 cells

**Fig 1:**
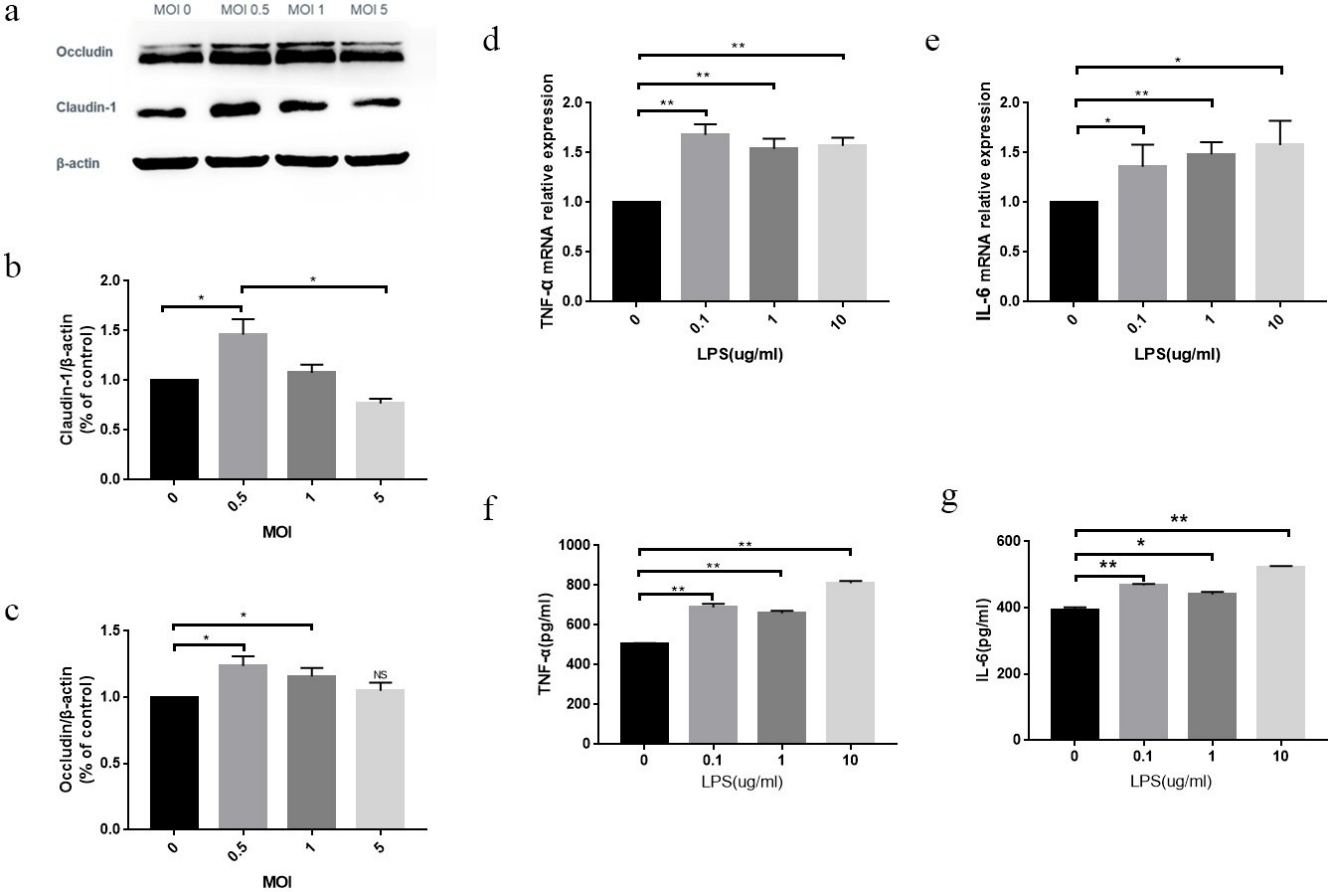
(a) Changes in the expression of claudin-1 (b) and occludin (c) proteins detected by WB. Data are presented as the mean ± SD. four independent experiments were performed in duplicate. *P < 0.05 vs. control group. RT-qPCR detection of TNF-α (d) and IL-6 (e) RNA expression in cells with different concentrations of LPS. Data are presented as the mean ± SD. three independent experiments were performed in duplicate. *P < 0.05 vs. control group, **P < 0.01 vs. control group. ELISA was used to detect the expression of TNF-α (f) and IL-6 (g) when different concentrations of LPS interfered with cells. Data are presented as the mean±SD. three independent experiments were performed in duplicate. *P < 0.05 vs. control group, **P < 0.01 vs. control group.

The expression of claudin-1 protein in the MOI of 0.5 group was elevated compared to the MOI of 0 group. The expression of a MOI of 1 and MOI of 5 was decreased compared to a MOI of 0 (P < 0.05; Fig 1A and 1B). The expression of occludin protein was elevated in each group compared to the MOI of 0 group. The expression of occludin in the MOI of 1 and MOI of 5 groups was lower than the MOI of 0.5 group (P < 0.05; Fig 1A and 1C).

### The appropriate concentration of LPS was 0.1–10 μg/ml

When the LPS concentration exceeded 0.1 μg/ml, the levels of TNF-α (Fig 1D) and IL-6 (Fig 1E) RNA expression increased compared with the control group (P < 0.05). ELISA findings showed that when the LPS concentration exceeded 0.1 μg/ml, TNF-α (Fig 1F) and IL-6 (Fig 1G) protein expression increased compared to the control group (P < 0.05).

### Therapeutic effects of LP on Caco2 cell monolayer membrane barrier injury induced by LPS (1 μg/ml)

**Fig 2:**
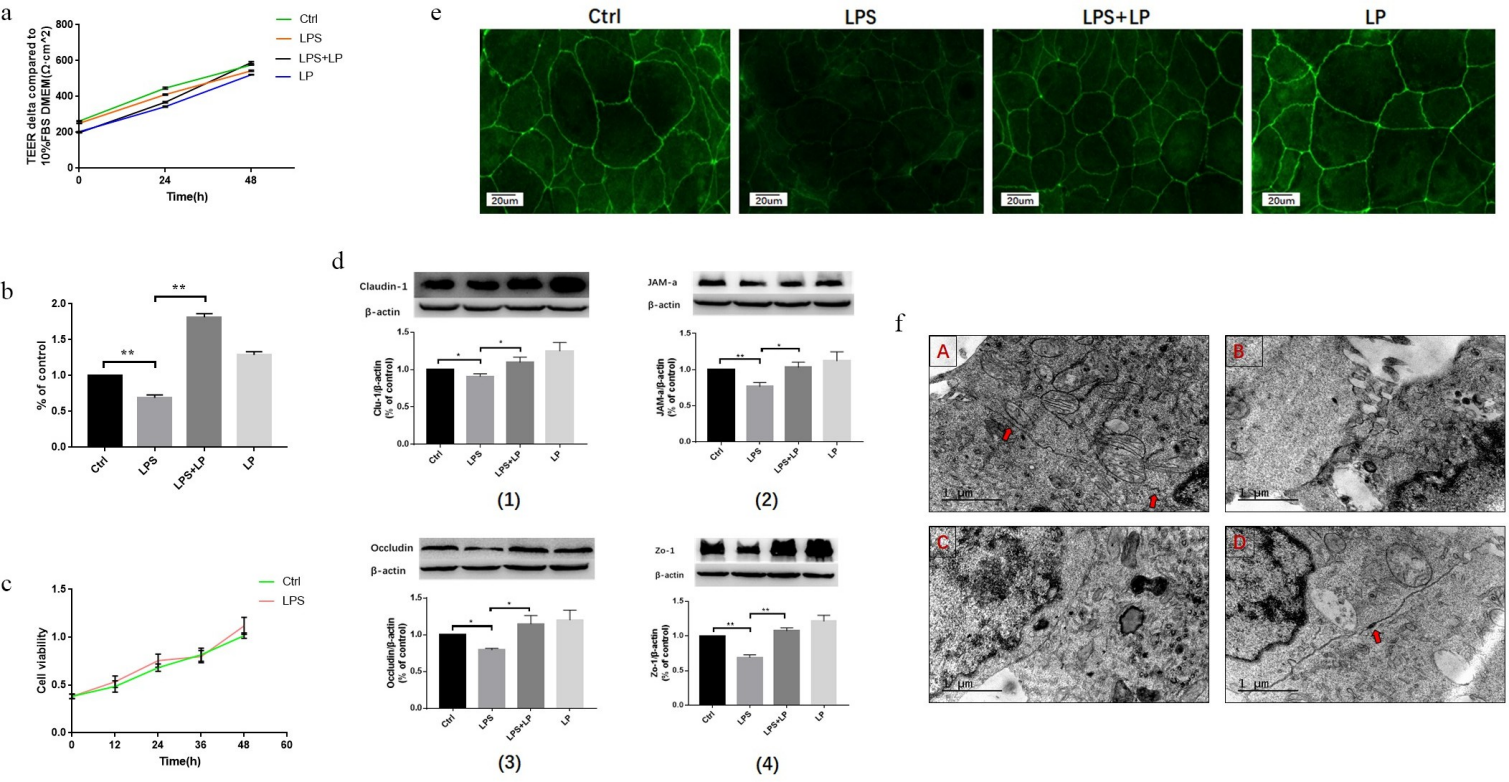
Measurement of TEER at 0, 24, and 48 h by each cell resistance meter (a) and TEER changes of each group at 24–48 h (b), Data are presented as the mean ± SD. three independent experiments were performed in duplicate. **P < 0.01 LPS + LP group vs. LPS group. (c) Effect of LPS treatment for 48 h on cell viability. (d) WB detection of claudin-1, JAM-a, occludin, and Zo-1 expression levels. Data are presented as the mean ± SD. four independent experiments were performed in duplicate. *P < 0.05 LPS + LP group vs. LPS group, **P < 0.01 LPS + LP group vs. LPS group. (e) Immunofluorescence detection of ZO-1 tight junction protein expression and distribution. three independent experiments were performed in duplicate. (f) Changes in electron microscopy-observed tightly connected cellular ultrastructure between groups. Arrows indicate the tight connection of Caco2 cells in each group A: Ctrl group, B: LPS group, C: LPS + LP group, D: LP group. three independent experiments were performed in duplicate.

TEER gradually increased over time (Fig 2A). To detect a difference in transmembrane electrical impedance between the LPS + LP group and the other groups after the addition of LP for 24 h, the difference at 24 and 48 h post-exposure was assessed. The TEER level in the LPS + LP group was significantly increased compared with the LPS group (P < 0.01; Fig 2B). In the previous experiment, the duration of LPS treatment of the cells was 24 h. To identify the cytotoxic effects of LPS treatment over 48 h, the control and 48-h LPS intervention groups were analyzed using the CCK-8 method. No significant difference in cytotoxicity was observed between the two groups. This finding indicated that LPS treatment of the cells for 48 h had no toxic side effects (Fig 2C).

Claudin-1, JAM-a, occludin, and ZO-1 proteins were increased in the LPS + LP group compared to the LPS group. LP promoted expression of claudin-1, JAM-a, occludin, and ZO-1 proteins in LPS-treated cells (P < 0.05; Fig 2D).

ZO-1 protein was highly distributed in a uniform pattern without interruption at the cell-cell surface interface of the control and LP groups. The ZO-1 protein fluorescence intensity was weakened in the LPS group, the cell interaction was incomplete, and the interruption was visible. In the LPS + LP group, the cell interaction was not interrupted. Compared with the LPS group, the fluorescence intensity was stronger, but still weaker than the control and LP groups (Fig 2E).

The junction structure was normal in the control group; the TJ structure is indicated by the arrow in Fig 2f A. Abnormal tightly connected structures were observed in the LPS group (Fig 2f B) and the connection between cells was incomplete. In addition, the intercellular space increased, the intracellular glycogen particles increased, and the intracellular organelle structure was unclear. Cellular TJ integrity was improved in the LPS + LP group (Fig 2f C) compared with the LPS group. The intracellular rough endoplasmic reticulum and other structures were visible. The LP group (Fig 2f D) was similar to the control group, with normal intercellular connections and a tightly connected ultrastructure was noted.

### LP-mediated effects on Caco2 monolayer membrane barrier damage induced by LPS (1 μg/ml) was achieved through cAMP-PKA regulation

**Fig 3:**
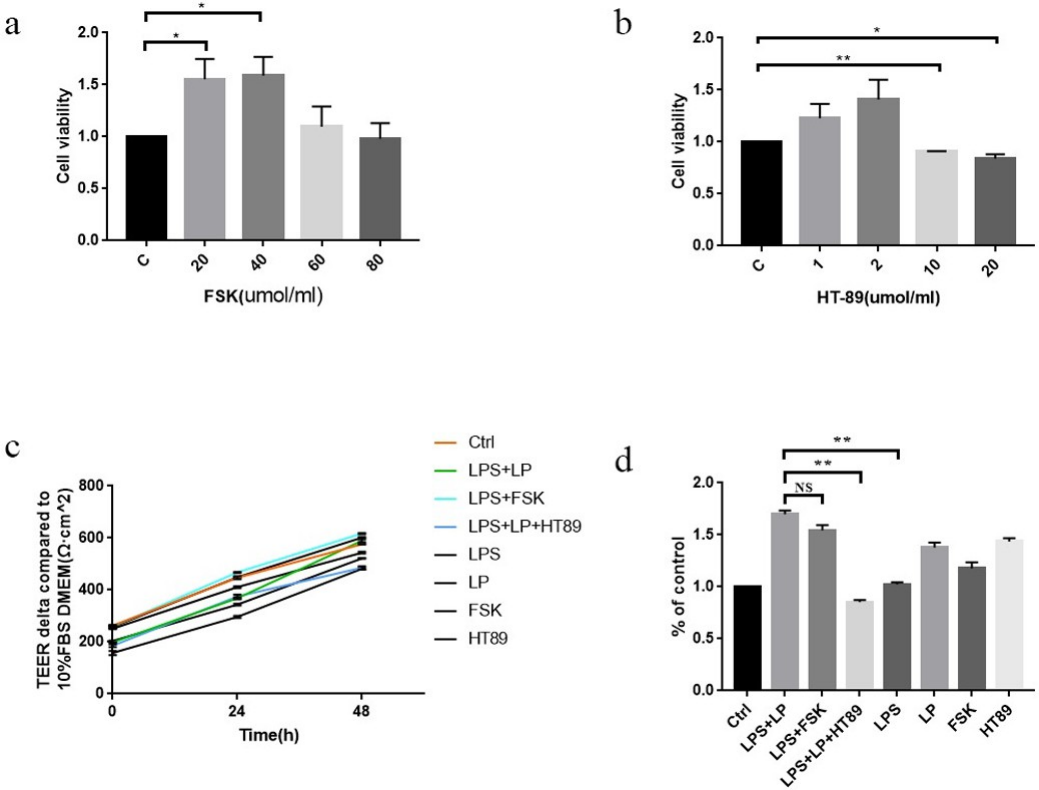
FSK toxicity on cells at different concentrations (a) in the experiment. Data are presented as the mean ± SD. five independent experiments were performed in duplicate.*P < 0.05 vs. control group. Toxicity of different concentrations of HT89 (b) on cells. Data are presented as the mean ± SD. three independent experiments were performed in duplicate. *P < 0.05 vs. control group, **P < 0.01 vs. control group. (c)-(d) TEER changes in each group at 0, 24, 48, and 24–48 h. Data are presented as the mean ± SD. three independent experiments were performed in duplicate. **P < 0.01 vs. control group or LPS + LP + HT89 group vs. LPS + LP group.

As the FSK concentration increased, cell viability was initially observed to have an increasing trend followed by a decreasing trend. Viability gradually increased at 0, 20 μmol/ml, and 40 μmol/ml (P < 0.05), and gradually decreased at higher concentrations (60 and 80 μmol/ml), although the difference was not statistically significant (Fig 3A).

As the concentration of HT89 increased, cell viability initially showed a trend of increased viability followed by a decrease. In the control group, treatment with 1 and 2 μmol/ml stimulated a gradually increasing viability trend, although the difference was not statistically significant. These findings confirmed that there was no inhibitory effect on the cells. At 10 and 20 μmol/ml cell viability gradually decreased, and the inhibitory effect on the cells was detectable (P < 0.05; Fig 3B).

TEER gradually increased over time (Fig 3C). Differences in TEER changes between groups after FSK (20 μmol /ml) and HT89 (2 μmol/ml) intervention at 24 and 48 h was analyzed and there was no significant difference in TEER levels between the LPS + FSK and the LPS + LP groups, though the TEER in the LPS + LP + HT89 group was significantly lower than the LPS + LP group (P < 0.01) (Fig 3D).

**Fig 4:**
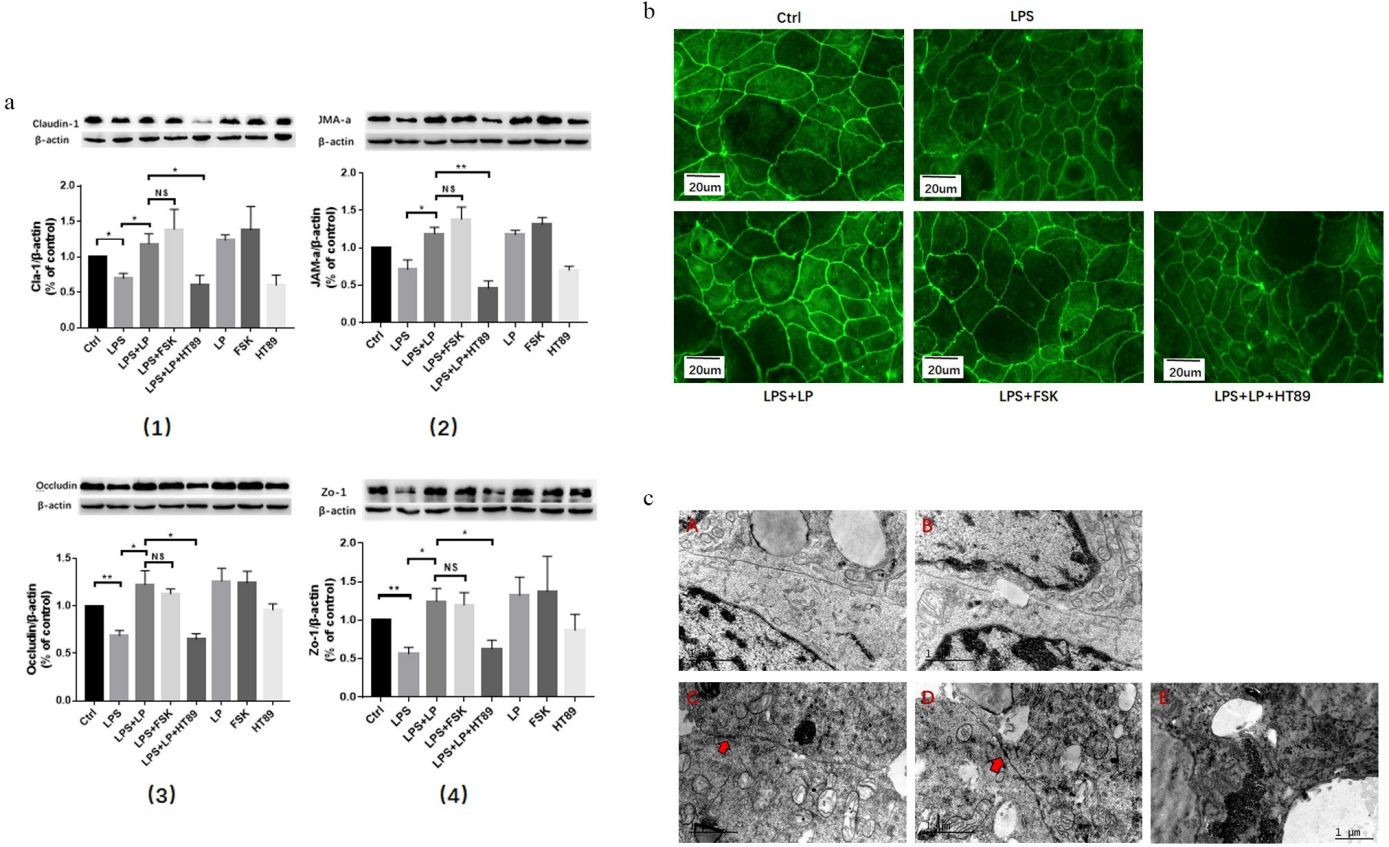
(a) WB detection of tight junction protein (claudin-1, JAM-a, occludin, and Zo-1) expression. Data are presented as the mean ± SD. three independent experiments were performed in duplicate. *P < 0.05 LPS + LP + HT89 group vs. LPS + LP group or LPS + LP group vs. LPS group. **P < 0.01 LPS + LP + HT89 group vs. LPS + LP group. (b) Immunofluorescent detection of the expression and distribution of the tight junction protein, ZO-1. three independent experiments were performed in duplicate. **(c)** Electron microscopy-observed changes of the tightly connected ultrastructure of cells between groups. Arrows indicate the tight connection of Caco2 cells in each group. A: Ctrl group, B: LPS group, C: LPS + LP group, D: LPS + FSK group, E: LPS + LP + HT89 group. three independent experiments were performed in duplicate.

The TJs proteins were more highly expressed in the LPS + LP group compared to the LPS group (P < 0.05). No statistically significant difference in protein expression between the LPS + FSK and LPS + LP groups was observed. The LPS + LP + HT89 group exhibited significantly lower TJ protein expression than the LPS + LP group (P < 0.05; Fig 4A).

ZO-1 protein in the LPS group was weaker than the control group, and the cell surface connection was interrupted. ZO-1 protein in the control, LPS + LP, and LPS + FSK groups was distributed along the cell-cell surface interaction with a uniform distribution, no interruption, and a strong fluorescence signal. Fluorescence intensity of ZO-1 in the LPS + LP + HT89 group was weaker compared with the LPS + LP group and the cell connection was interrupted (Fig 4B).

In the control group (Fig 4c A), a complete TJ ultrastructure formed between cells, and cell connections were not interrupted. In the LPS group (Fig 4c B), discontinuous breaks occurred along the sites of the intercellular interaction, and a tight connection did not form. Compared with the LPS group, there were no discontinuities in tight connections between cells; the tight connection structure was complete and no abnormal tight connection structures were visible in the LPS + LP group (Fig 4c C). The LPS + FSK group (Fig 4c D) showed a similar tightly connected structure as the LPS + LP group. In the LPS + LP + HT89 group (Fig 4c E), the intercellular junction structure was not apparent; the TJ structure was abnormal with many intracellular glycogens and LP action was antagonized by HT89.

## Discussion

Sepsis causes thousands of deaths each year, and as our understanding of intestinal diseases grows, the role of microorganisms in intensive care units and intestinal functions becomes clearer [22].

We selected the *in vitro* sepsis model based on a study by Hong et al. (2016) in which LPS was used to induce the model in Caco2 cells and NEC rats [23]. Hong et al. (2016) then co-treated the models with intestinal *Bifidobacterium*, which confirmed the protective intestinal barrier function, and proved the feasibility of the approach. The LP was added to the Caco2 cell culture system to build a micro-ecosystem. The same method was used in experiments by Gaiani et al. [24]. Our results showed that the appropriate LPS concentration range was 0.1–10 μg/ml in our model. We chose 1 μg/ml from this range as the experimental concentration based on a review of the literature [8].

A tight connection is formed when the TEER is > 150 Ω/cm² [25, 26]. ZO-1 is involved in maintaining and regulating the barrier function of the TJ structure epithelial cells and is the most basic protein in this structure [27, 28]. Western blot and fluorescence experiments confirmed that ZO-1 protein expression increased after LP intervention.

The integrity of TJ structures is a key factor in intestinal cell barrier function. Our electron microscopy results confirmed that after LP treatment, the TJ structure between the cells was more complete, which is consistent with the results of a previous study [29]. In addition, electron microscopy revealed that cells treated with LPS exhibited increased lipid droplets (Fig 3.4.2 A and B) and glycogen particles (Fig 3.4.2 C). Studies have shown that apoptotic cell death is positively correlated with the role of antioxidants and the expression of adipogenic genes [30]. The accumulation of glycogen can cause cell death in some cell types [31, 32]. Addition of LP improves barrier function [33]; however, the mechanism by which LP positively affects apoptosis, lipid metabolism, and carbohydrate metabolism warrants further investigation.

Many studies have shown that cAMP triggers upregulation of TJ-related protein levels, increases the transcellular impedance value of epithelial cells, and thereby strengthens the TJs between cells. In addition, PKA is activated by cAMP, which has a regulatory effect on TJ assembly and the opening of epithelial side pathways [34]. By comparing the experimental results of LP intervention, LP and FSK were shown to have the same role in stimulating cAMP to regulate PKA signaling. The effect of LP was antagonized by HT89, indicating that LP regulates TJ structure through PKA signaling, and clarifies LP regulation of the intestinal mucosal barrier function through the cAMP-PKA pathway. Zhou et al. (2012) reported that LP prevents the destruction of TJ proteins in biliary obstruction by activating the PKC pathway and plays a role in protecting the intestinal barrier [35].

With respect to molecular mechanisms, our experiments had some limitations. Our experiments did not investigate the downstream signaling molecules of the cAMP-PKA pathway, thus limiting our potential interpretation of the downstream mechanisms by which cAMP-PKA signaling mediates LP action on epithelial cells. In the experiment conducted by Jiang et al. (2021), intestinal porcine cells were incubated with 0 or 1 × 108 CFU per well LP for 4 h, then these cells were challenged with 0 or 1 × 108 CFU per well ETEC K88 for 2 h. It was proved that pretreatment with LP downregulated NFκB concentration. Silencing experiments confirmed that the protective effect of LP may be realized through inhibition of MAPK and NFκB pathways [36]. A number of experiments have also shown that LP reduces inflammation and increase tight junction protein expression to protect host cells by inhibiting the NFκB pathway [37–41]. Studies have shown that lactobacillus inhibits the upregulation of IL-6 and CXCL8 through TLR negative regulators (A20, Tollip, SIGIRR, and IRAKM), P38 MAPK and P65 NF-κB signaling pathways, thus playing a protective role in regulating immunity [42, 43]. Therefore, whether the inhibition of NFκB by LP in LPS-mediated injury is regulated through the cAMP-PKA pathway will be explored as an idea in future experiments.

Our model showed that LP regulates cell metabolism to resist pathogens by maintaining the integrity of TJ epithelium and reducing LPS-induced TJ damage. Our research also showed that LP protected the integrity of the TJ structure by regulating cAMP and PKA cell signaling molecules, thereby improving the intestinal mucosal barrier function. Elucidating the mechanism of probiotics acting on the intestinal barrier will promote application in clinical treatment and prevention. In addition, further animal studies are needed to determine how LP protects the intestinal barrier both before and after onset. In future studies, we will conduct an in-depth analysis on the mechanism underlying intestinal LP function to expand scientific knowledge of LP influence in the treatment of sepsis.

## Conclusion

Our experimental findings showed that LP exhibits protective and regulatory effects on TJ structure in the Caco2 cell model induced by LPS, and confirmed that LP exerts a regulatory role through the cAMP-PKA pathway.

## Supporting information

S1 File (PDF)
