# Supplementary material for: Lactobacillus plantarum improves LPS-induced Caco2 cell line intestinal barrier damage via cyclic AMP-PKA signaling
Source: PLoS One. 2022 May 31;17(5):e0267831. doi: 10.1371/journal.pone.0267831 (PMC9154120; doi:10.1371/journal.pone.0267831)

Fig.1.a

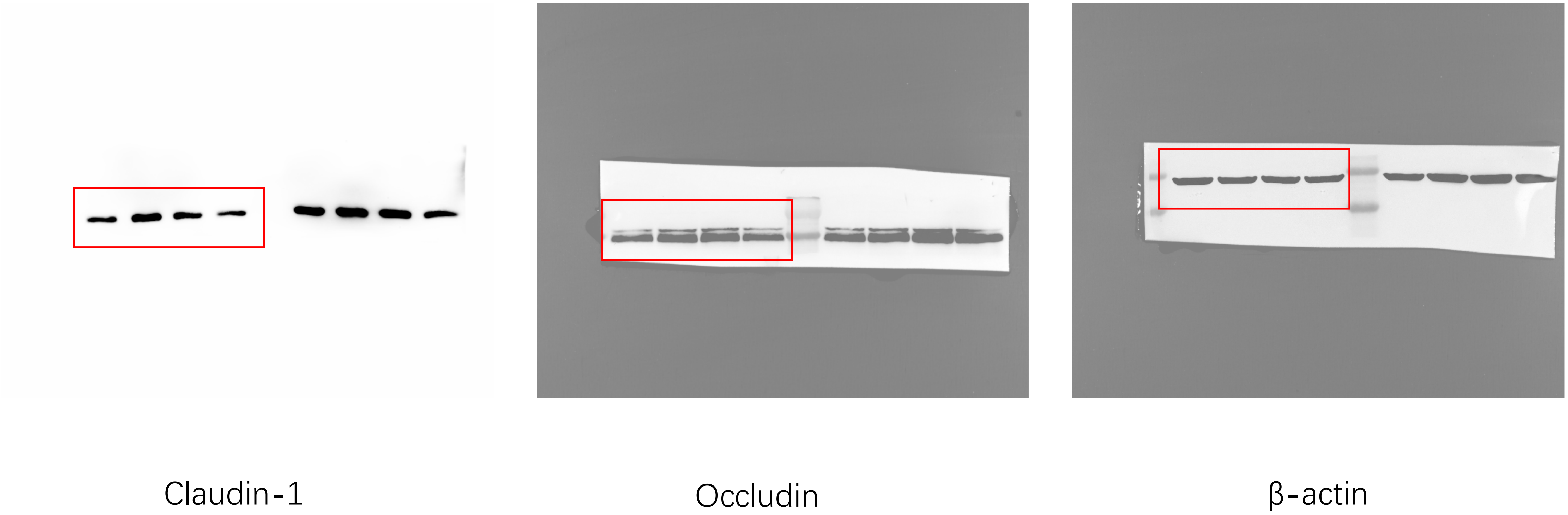

Fig.2.d

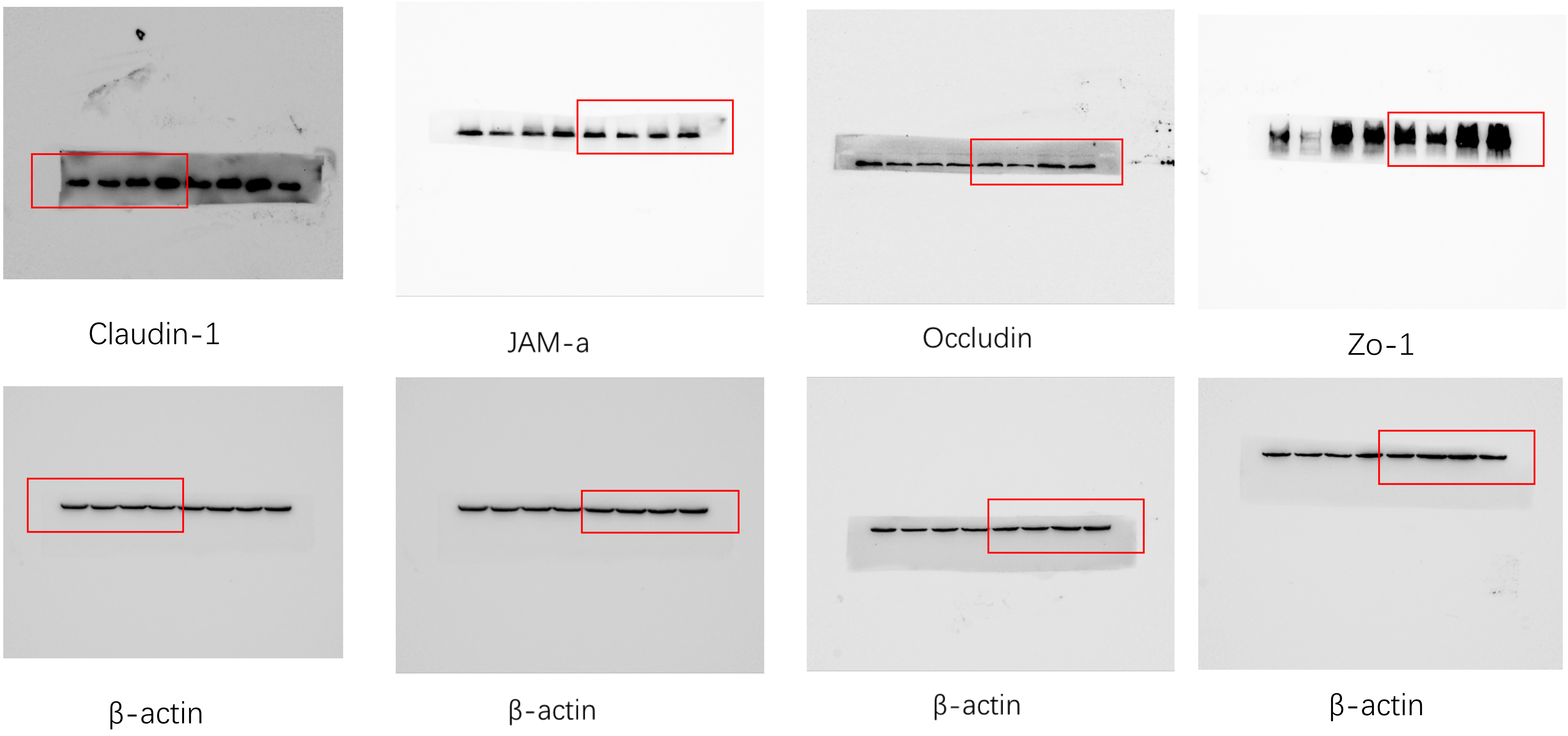

Fig.4.a

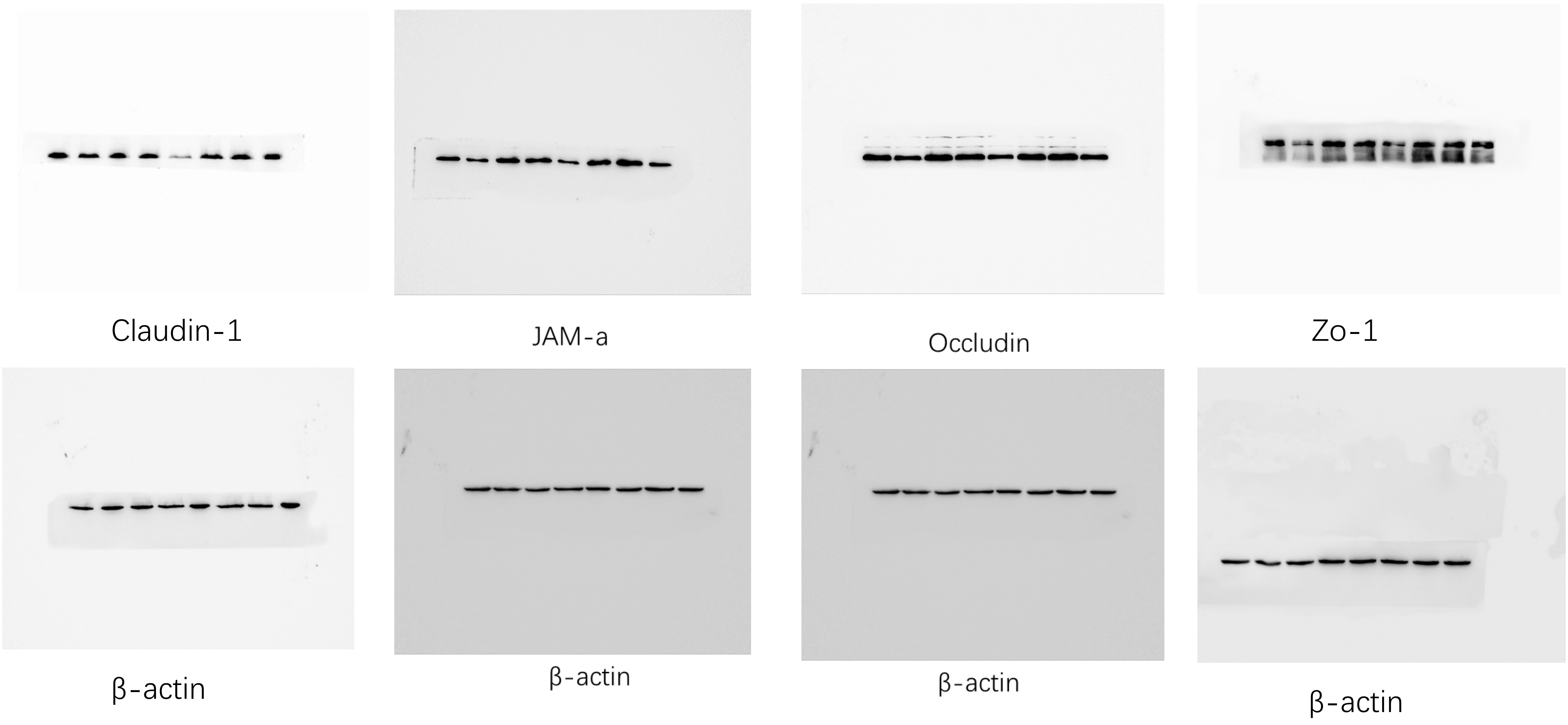

Supplement: S1 File — (PDF) [file pone.0267831.s001.pdf]
